# Endorectal Ultrasonography and Pelvic Magnetic Resonance Imaging Show Similar Diagnostic Accuracy in Local Staging of Rectal Cancer: An Update Systematic Review and Meta-Analysis

**DOI:** 10.3390/diagnostics12010005

**Published:** 2021-12-21

**Authors:** Gaetano Luglio, Gianluca Pagano, Francesca Paola Tropeano, Eduardo Spina, Rosa Maione, Alessia Chini, Francesco Maione, Giuseppe Galloro, Mariano Cesare Giglio, Giovanni Domenico De Palma

**Affiliations:** 1 Endoscopic Surgery Unit, Department of Medical and Surgical Gastrointestinal Disease, Federico II University of Naples, 80131 Naples, Italy; gaetano.luglio@unina.it (G.L.); fpt.tropeano@gmail.com (F.P.T.); spinaed@gmail.com (E.S.); rosamaione95@libero.it (R.M.); alessiachini@hotmail.it (A.C.); francescomaione79@gmail.com (F.M.); giuseppegalloro60@gmail.com (G.G.); giovanni.depalma@unina.it (G.D.D.P.); 2 Division of HPB, Minimally Invasive and Robotic Surgery, Federico II University of Naples, 80131 Naples, Italy; mariano.giglio@hotmail.it

**Keywords:** rectal cancer, staging, endorectal ultrasonography, pelvic magnetic resonance imaging

## Abstract

Background: Endorectal Ultrasonography (EUS-ERUS) and pelvic magnetic resonance imaging (MRI) are world-wide performed for the local staging of rectal cancer (RC), but no clear consensus on their indications is present, there being literature in support of both. The aim of this meta-analysis is to give an update regarding the diagnostic test accuracy of ERUS and pelvic MRI about the local staging of RC. Materials and methods: A systematic literature search from November 2020 to October 2021 was performed to select studies in which head-to-head comparison between ERUS and MRI was reported for the local staging of rectal cancer. Quality and risk of bias were assessed with the QUADAS-2 tool. Our primary outcome was the T staging accuracy of ERUS and MRI for which pooled accuracy indices were calculated using a bivariable random-effects model. In addition, a hierarchical summary receiver operating characteristic curve (hSROC) was created to characterize the accuracy of ERUS and MRI for the staging of T and N parameters. The area under the hSROC curve (AUC_hSROC_) was determined as a measure of diagnostic accuracy. Results: Seven studies and 331 patients were included in our analysis. ERUS and MRI showed a similar accuracy for the T staging, with AUC_hSROC_ curves of 0.91 (95% C.I., 0.89 to 0.93) and 0.87 (95% C.I., 0.84 to 0.89), respectively (*p* = 0.409). For T staging, ERUS showed a pooled sensitivity of 0.82 (95% C.I. 0.72 to 0.89) and pooled specificity of 0.91 (95% C.I. 0.77–0.96), while MRI had pooled sensitivity and specificity of 0.69 (95% C.I. 0.55–0.81) and 0.88 (95% C.I. 0.79–0.93), respectively. ERUS and MRI showed a similar accuracy in the N staging too, with AUC_hSROC_ curves of 0.92 (95% C.I., 0.89 to 0.94) and 0.93 (95% C.I., 0.90 to 0.95), respectively (*p* = 0.389). Conclusions: In conclusion, ERUS and MRI are comparable imaging techniques for the local staging of rectal cancer.

## 1. Introduction

Rectal cancer (RC) occupies a prominent position among the most common neoplasms all over the world. In fact, the last report of the Global Cancer Observatory (GCO) revealed 732,210 new diagnoses and 339,022 deaths in the last year, with Asia and Europe the most affected continents [1].

The treatment pathway of RC depends upon the effective coordination between healthcare professionals: the presence of a multidisciplinary team has to be made of oncologists, colorectal surgeons, radiotherapists, radiologists, pathologists and endoscopists. This is so crucial as the oncological outcome can be compromised by the absence of any one of these figures [2]. In fact, a retrospective analysis showed that unsuccessful multidisciplinary discussion was one of the predictive factors for positive resection margins, as well as the absence of radiotherapy [3].

Subsequent to digital rectal examination, a colonoscopy is compulsory to confirm or disconfirm the clinical suspect of RC. Moreover, the endoscopists can benefit from the use of confocal laser endomicroscopy (CLE) for an in vivo and non-invasive assessment of lesions’ vascular microarchitecture [4,5,6]. Further molecular investigations can be performed in order to predict the response to neoadjuvant chemoradiotherapy in selected cases [7].

Once diagnosed, RC requires to be properly classified and staged [8,9]. RC classification as low–middle–high RC (according to its distance from the anal verge, 5–10–15 cm, respectively) and intraperitoneal/extraperitoneal is crucial for further management steps: intraperitoneal RC can be treated as colon cancer while extraperitoneal RC requires different staging and therapeutic procedures. Not least, determining the extension of the disease through the intestinal wall and metastatic lymph nodes requires expertise and the proper diagnostic techniques. Endorectal ultrasonography (ERUS) and pelvic magnetic resonance imaging (MRI) play a crucial role in RC staging and results regarding their diagnostic accuracy are quite contrasting. In fact, while a meta-analysis showed the superiority of ERUS in determining local invasion compared to MRI, a systematic review found that both techniques were equivalent [10,11]. Computed tomography (CT) of the chest and the abdomen is preferred to assess the presence of metastasis, thus completing the clinical staging. Another aspect that deserves considerable attention in preoperative staging of RC is the invasion of the circumferential resection margin (CRM), which is, actually, the mesorectal fascia enclosing the mesorectum.

The aim of this meta-analysis is to give an update regarding the diagnostic test accuracy of ERUS and pelvic MRI about the local staging of RC.

## 2. Materials and Methods

### 2.1. Search Strategy

A systematic literature search of the MEDLINE, Embase and Web of Science databases was carried out using the following search terms: rectal cancer, magnetic resonance imaging, endorectal ultrasonography, local staging, accuracy (electronic search strategy as Supplementary Material), with no language or publication status limitations. Searches were cross-referenced and extended on MEDLINE using the related articles function. The reference list of retrieved articles was also used to identify additional eligible studies. The first search was carried out on 10 November 2020, and the last search was undertaken on 10 October 2021.

### 2.2. Study Selection

To be eligible for the final analysis, studies had to (i) include patients over 18 years of age undergoing rectal cancer surgery with curative intent; (ii) compare patients undergoing both ERUS and pelvic MRI for RC clinical staging; (iii) consider histopathological examination as reference standard. Studies were excluded if they were (i) review articles, case reports, laboratory studies or letters; (ii) unpublished data from meeting abstracts; (iii) lacking essential information for the calculation of outcomes. Two reviewers (G.L. and G.P.) independently assessed the retrieved references at title and abstract level to identify potential eligible studies. Any conflict was resolved by a third reviewer (F.P.T.) until a consensus was reached. The full text of these studies was then retrieved for further analysis.

### 2.3. Data Extraction, Quality and Risk of Bias Assessment

Two authors (M.C.G. and G.P.) independently extracted or calculated relevant data from each included study by completing an electronic database with the following information: first author, year of publication, number of patients involved, study design, type of imaging technique, reference standard. True positives, false positives, true negatives and false negatives for both ERUS and MRI were calculated and retrieved by the two authors for each T and N stage.

In adherence to Cochrane Collaboration recommendation, quality and risk of bias assessment was performed with the QUADAS-2 tool. This consists of four key domains: patient selection, index test, reference standard, flow and timing. Each domain is assessed in terms of risk of bias and the first three in terms of concerns regarding applicability. Signaling questions are included to assist in judgements about risk of bias [12].

### 2.4. Data Synthesis and Statistical Analysis

The primary outcome of this meta-analysis was the T staging accuracy of ERUS and MRI. Sensitivity, specificity, positive predictive value (PPV), negative predictive value (NPV), positive likelihood ratio (LR), negative LR were calculated as summaries of the diagnostic performances for each study. Pooled accuracy indices were then calculated using a bivariable random-effects model [13]. In addition, a hierarchical summary receiver operating characteristic curve (hSROC) was created to characterize the accuracy of ERUS and MRI for the staging of T and N parameters [14]. The area under the hSROC curve (AUC_hSROC_) was determined as a measure of diagnostic accuracy.

ERUS and MRI staging accuracy for each staging parameter was compared using meta-regression analysis. The heterogeneity between the studies was assessed using the *Q* value and the inconsistency index (*I*^2^) [15]. Low, moderate and high statistical heterogeneity were identified by *I*^2^ values of 25, 50 and 75%, respectively.

The Review Manager Calculator tool (RevMan, version 5.3; The Cochrane Collaboration, The Nordic Cochrane Centre, Copenhagen, Denmark) was used to calculate accuracy values (sensitivity, specificity, PPV, NPV, positive and negative LR) from the data available in the selected studies. Other analyses were performed using STATA^®^ 12 statistical software (StataCorp LP, College Station, TX, USA) and R version 3.6.1 (2019, The R Foundation for Statistical Computing).

## 3. Results

### 3.1. Study Selection

**Figure 1:**
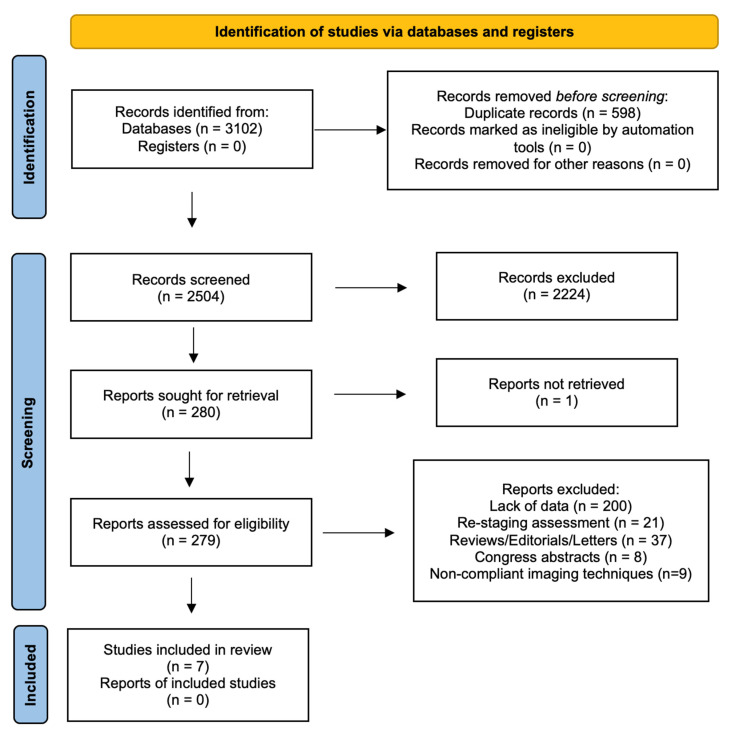
PRISMA flow diagram.

**Figure 2:**
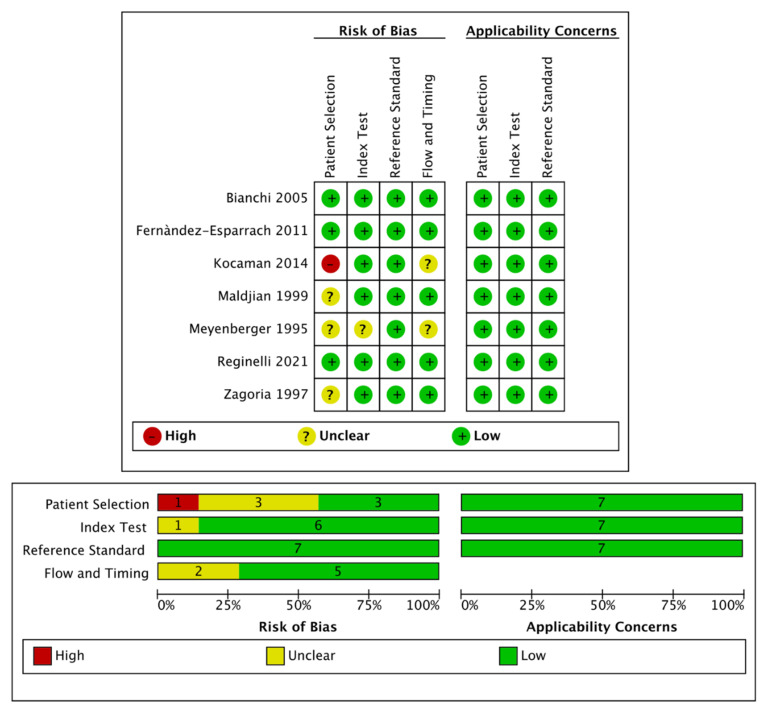
Quality assessment of the included studies via QUADAS-2 tool.

The search of MEDLINE, Embase and Scopus^®^ databases revealed a total of 3102 citations; 598 of these were found to be duplicated and were therefore removed. Of the remaining 2504 studies, 2224 were excluded because they did not meet the inclusion criteria after review of the title or abstract and one more was excluded for another reason. The full text of each of the remaining 279 articles was examined in more detail. This led to the removal of a further 272 studies, leaving seven studies that were consequently included in the meta-analysis. The Preferred Reporting Items for Systematic Reviews and Meta-Analyses (PRISMA) flow diagram is shown in Figure 1. Quality assessment of the included studies with QUADAS-2 graphs is reported in Figure 2.

### 3.2. Study Characteristics

The seven studies included involved a total of 331 patients [16,17,18,19,20,21,22]. Two studies were retrospective [21,22] and five were prospective [16,17,18,19,20]. All of the patients involved were diagnosed with rectal cancer and underwent local staging with both ERUS and pelvic MRI before surgery. Histopathological examination of the surgical specimen was indicated as a reference standard test. Relevant characteristics of the included studies are summarized in Table 1.

### 3.3. T Staging, ERUS vs. MRI

**Figure 3:**
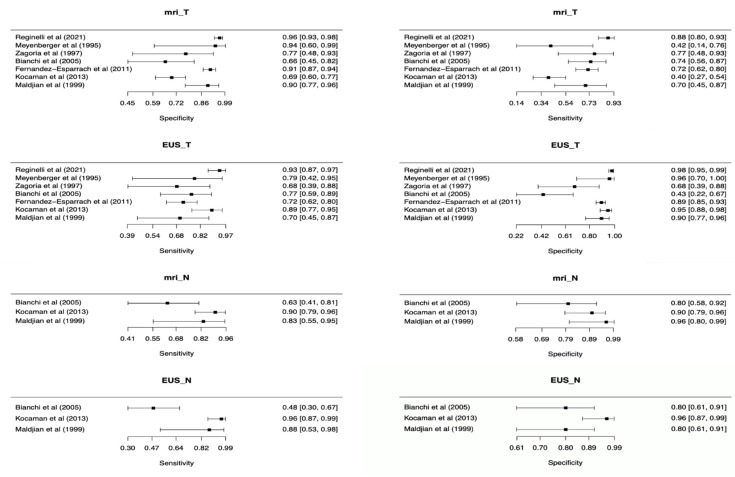
Forest plots of included studies showing overall T and N parameters’ sensitivities and specificities in rectal cancer staging.

For T staging, ERUS showed a pooled sensitivity of 0.82 (95% C.I. 0.72 to 0.89) and pooled specificity of 0.91 (95% C.I. 0.77–0.96), while MRI had pooled sensitivity and specificity of 0.69 (95% C.I. 0.55–0.81) and 0.88 (95% C.I. 0.79–0.93), respectively. Additional pooled measures of diagnostic performances of ERUS and MRI are reported in Table 2 and Forest plots displaying overall sensitivity and specificity for each included study in Figure 3.

**Figure 4:**
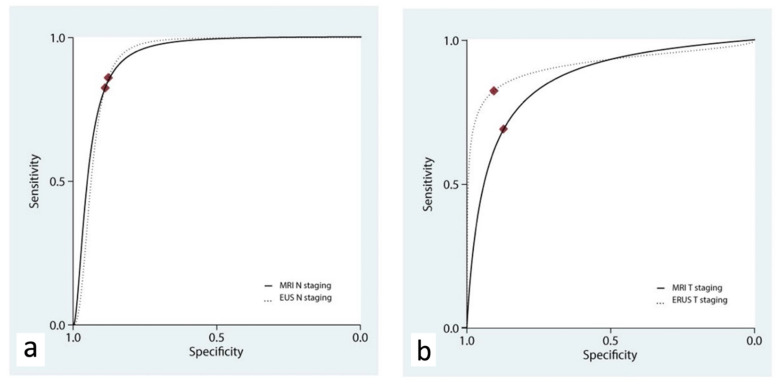
Hierarchical summary receiver operating characteristic curve (hSROC) characterizing the accuracy of ERUS and MRI for the staging of N (**a**) and T (**b**) parameters.

ERUS and MRI showed a similar accuracy for the T staging, with AUC_hSROC_ curves of 0.91 (95% C.I., 0.89 to 0.93) and 0.87 (95% C.I., 0.84 to 0.89), respectively (*p* = 0.409, Figure 4a and Table 3).

Table 2 reports the ERUS and MRI performance for each single T stage. ERUS outperformed MRI in all but one T stage, with remarkable difference in T1 tumors. On the other hand, T4 RC can benefit from MRI which, at this stage, is connoted by higher sensitivity (0.75, 95% C.I. 0.37–0.94 vs. 0.62, 95% C.I. 0.25–0.88) and slightly lower specificity (0.95, 95% C.I. 0.90–0.97 vs. 0.98, 95% C.I. 0.95–0.99).

### 3.4. N Staging, ERUS vs. MRI

The accuracy of ERUS and MRI for N staging was assessed in three studies [18,19,21]. ERUS had a pooled sensitivity of 0.83 (95% C.I. 0.45–0.96) and a pooled specificity of 0.88 (95% C.I. 0.73–0.95), while MRI had a pooled sensitivity of 0.82 (95% C.I. 0.64–0.92) and a pooled specificity of 0.89 (95% C.I. 0.79–0.95). ERUS and MRI showed a similar accuracy in the N staging, with AUC_hSROC_ curves of 0.92 (95% C.I., 0.89 to 0.94) and 0.93 (95% C.I., 0.90 to 0.95), respectively (*p* = 0.389, Figure 4b).

## 4. Discussion

The present meta-analysis concluded that ERUS and MRI are, in general, comparable imaging techniques for the local staging of rectal cancer. Going into details, ERUS appears to be more sensitive than MRI for the local staging of all but T4 lesions in which this trend is inverted; as for nodal involvement, ERUS and MRI are almost equivalent.

The setting in which our meta-analysis lies is quite controversial. In fact, guidelines from societies most referred to as references around the world are not on the same thinking line regarding this issue. The latest version (2.2021) of the National Comprehensive Cancer Network (NCCN) guidelines conclude that MRI is the preferred technique, but it may not be required for local staging if the tumor is known to be a definite cT1 and can be replaced by ERUS in the case of patient-specific contraindications. By contrast, the American Society for Gastrointestinal Endoscopy recommend ERUS for locoregional staging to guide therapy, while a more moderate position is held by the European Society of Medical Oncology, which stand for the use of ERUS or MRI in early T staging, with MRI preferred for N staging [23,24].

The role of pelvic MRI in RC staging has been deeply investigated by the MERCURY study group. Between January 2002 and October 2003, consecutive patients with biopsy-proven RC were enrolled in this observational study that aimed to assess the diagnostic accuracy of preoperative MRI in predicting curative resection in RC surgery. Of the 679 potentially eligible patients, complete pathology and MRI data were available for 408 patients who constituted the final sample.

Results showed that MRI predicted clear margins in 349 patients. All of these underwent surgery and 327 of them were confirmed to have clear margins at histopathology (94%), resulting in a technique specificity value of 92%. Moreover, 311 patients underwent primary surgery. The accuracy for predicting a clear margin was 91% with a negative predictive value of 93% [25]. Five-year follow-up results regarding the prognostic relevance of MRI assessment of circumferential resection margin in RC were published [26]. Of the original sample size, complete histopathologic and radiologic data were available for 374 patients. MRI predicted a potentially involved CRM in 64 patients; of these, 32 of 64 relapsed and 38 died. The 5-year OS was 62.2% in patients with mrCRM clear compared with 42.2% in patients with predicted mrCRM involved. mrCRM involvement remained significant for poor OS on multivariate analysis. The 5-year DFS was 67.2% for patients with mrCRM clear compared with 47.3% for patients with mrCRM involved. The same dataset of the first MERCURY study allowed this multicentric study group also to demonstrate that their MRI data were almost equivalent to histopathologic results regarding the preoperative prediction of tumor invasion [27].

Therefore, the “MERCURY lesson” focuses on the accurate prediction of CRM by MRI. Moreover, it can be reproduceable in numerous centers to assess curative resections and alert the multidisciplinary team to the possibility of surgery failure, allowing patients to be selected for preoperative care.

The first large series regarding the accuracy of ERUS in preoperative staging of RC was published by Garcia-Aguilar et al. in which 545 patients were included for the final analysis [28]. Results from their retrospective study showed that the overall accuracy of ERUS in determining T stage was 69% with a positive predictive value of 72% and a negative predictive value of 93%. Node metastasis identification accuracy was evaluated in a sub-group of 238 patients who underwent radical surgery without preoperative radiation therapy, and the resulting value was of 64%.

During the last decades, several attempts to compare both techniques have been carried out with systematic reviews and meta-analysis. In particular, three of them are worth mentioning. The first work, by Kwok et al., is a systematic review in which 83 studies reporting data on 4879 patients were included [11]. A combination of CT, MRI and ERUS was used for preoperative staging of RC and histopathology as a referral standard. According to their results, ERUS demonstrated the highest sensitivity, specificity and accuracy in the assessment of wall penetration. MRI assessment of wall penetration had lower sensitivity, specificity and accuracy than ERUS but, when performed with an endorectal coil, the sensitivity, specificity and accuracy were similar to those of ERUS. As for nodal involvement assessment, MRI performed with an endorectal coil showed the highest sensitivity, specificity and accuracy, while ERUS had similar results to MRI overall.

A meta-analysis conducted by Bipat et al. [10] on 90 studies highlighted that regarding *muscularis propria* invasion ERUS and MRI had similar sensitivity estimates of 94%, while specificity for ERUS (86%) was significantly higher than that for MRI imaging (69%), indicating overstaging of T1 (or lower) tumors with this latter technique. Taking lymph node involvement into account, the resulting sensitivity estimates for ERUS and MRI were comparably low: 67% and 66%, respectively. Specificity values were also comparable: 78% for ERUS and 76% for MRI.

However, even though including a considerable amount of data, these two studies were burdened by remarkable limitations as the absence of a head-to-head comparison between patients and preoperative staging techniques.

Recently, Chan et al. conducted a well-designed meta-analysis to overcome the abovementioned issues [29]. In particular, their paper was based on six studies enrolling a total of 234 patients who underwent the same preoperative staging course with ERUS plus pelvic MRI and, for some of them, CT scan [16,17,18,19,20,21]. Imaging findings were compared to surgical specimen histopathology as a standard of reference. Data analysis revealed that ERUS was significantly superior to MRI in overall T staging with the two areas under the curve (AUC) of 0.87 and 0.82, respectively, while there were no differences between the two groups in overall N staging.

Nowadays, having proper local staging is of primary importance for the surgeon as different surgical options are available depending on the extent and location of the disease. In fact, the patient can benefit from less invasive techniques such as transanal local excision and transanal endoscopic microsurgery (TEM) as well as traditional methods such as total mesorectal excision (TME) or abdominoperineal resection (APR) [30]. Indications to local approaches are strict and rely on imaging.

Bearing this in mind, pelvic MRI has the ability to provide accurate images of the tissue structures in the mesorectum, including the mesorectal fascia, so as to provide information useful in the prediction of the CRM prior to radical surgery [31,32,33,34].

The choice of the appropriate surgical procedure is not the only staging-related concern to be considered. Indeed, once a patient receives a diagnosis of locally-advanced RC (stage II cT3-4 N0 or stage III N+), neoadjuvant chemoradiotherapy becomes a fundamental step before surgery. Additionally, as reported in an analysis conducted by Brown et al., incorrect preoperative treatment on the basis of either DRE, ERUS and MRI can have such an impact on the health system. As for the total costs incurred (procedure costs and costs resulting from incorrect preoperative treatment), the resource benefits that result from the use of MRI rather than DRE amount to GBP 67,164 and GBP 92,244 when MRI is used rather than ERUS. In addition, MRI correctly staged 86 patients, 47 more than DRE and 39 more than ERUS. In conclusion, in terms of cost-effectiveness, MRI dominates both DRE and ERUS [35].

This paper is not free of limitations. First, we compared two techniques that are extremely operator dependent for generating possible biases in their interpretation. Then, there was variation in the type of coil utilized as well as the field strength in the included research, which must be considered when depicting our findings. Finally, this meta-analysis has a very strict inclusion criteria, which is represented by the “head-to-head” comparison between patients who underwent both ERUS and pelvic MRI as imaging techniques for RC clinical staging, resulting in the inclusion of 331 patients; more research and a wider number of patients would almost certainly result in a more accurate evaluation and comparison of outcomes.

As well as the results of this meta-analysis, multidisciplinary discussion and a high level of expertise among healthcare providers are always required in pursuing the best work-up management and better outcomes in patients’ care.

## 5. Conclusions

In overall, ERUS and MRI are comparable imaging techniques for the local staging of rectal cancer in experts’ hands. While ERUS is more reliable for characterizing early stage lesions, both techniques are nearly identical in terms of nodal involvement detection.

## Figures and Tables

**Table 1:** Included studies characteristics.

Authors	Year	N. of Patients	Design	MRI	ERUS	Reference Standard
Meyenberger et al. [16]	1995	21	Prospective	1.5 T endorectal coil	Radial 7.5 MHz	Histopathology
Zagoria et al. [17]	1997	10	Prospective	1.5 T endorectal coil	Radial 7.5 MHz	Histopathology
Maldjian et al. [19]	2000	14	Prospective	1.5 T endorectal coil and body coil	Radial 7.5 MHz or 12 MHz	Histopathology
Bianchi et al. [18]	2005	49	Prospective	1 T body coil	7.5 MHz	Histopathology
Fernández-Esparrach et al. [20]	2011	90	Prospective	1.5 T or 3 T	Radial	Histopathology
Kocaman et al. [21]	2014	50	Retrospective	1.5 T phased array coil	Radial 7.5 MHz or 10 MHz	Histopathology
Reginelli et al. [22]	2021	97	Retrospective	1.5 T phased array coil	10–13 MHz	Histopathology

**Table 2:** Pooled measures of diagnostic performances.

	Sensitivity [95% C.I.]	Specificity [95% C.I.]	Positive LR [95% C.I.]	Negative LR [95% C.I.]
**ERUS T**	0.82 [0.72, 0.89]	0.91 [0.77, 0.96]	8.8 [3.2, 24.0]	0.19 [0.11, 0.34]
**MRI T**	0.69 [0.55, 0.81]	0.88 [0.79, 0.93]	5.6 [2.7, 11.6]	0.35 [0.21, 0.57]
**ERUS N**	0.83 [0.45, 0.96]	0.88 [0.73, 0.95]	6.7 [2.3, 19.9]	0.20 [0.04, 0.91]
**MRI N**	0.82 [0.64, 0.92]	0.89 [0.79, 0.95]	7.5 [3.5, 16.5]	0.20 [0.09, 0.45]
**ERUS T1**	0.78 [0.55, 0.91]	0.96 [0.88, 0.99]	19.4 [5.8, 64.7]	0.23 [0.10, 0.53]
**MRI T1**	0.47 [0.10, 0.87]	0.98 [0.94, 1.00]	27.6 [7.3, 104.7]	0.54 [0.21, 1.39]
**ERUS T2**	0.70 [0.51, 0.84]	0.92 [0.83, 0.97]	9.4 [3.3, 26.6]	0.32 [0.17, 0.60]
**MRI T2**	0.61 [0.39, 0.79]	0.86 [0.63, 0.96]	4.3 [1.1, 16.5]	0.46 [0.23, 0.89]
**ERUS T3**	0.92 [0.83, 0.97]	0.79 [0.64, 0.89]	4.4 [2.4, 8.1]	0.10 [0.04, 0.25]
**MRI T3**	0.81 [0.60, 0.92]	0.77 [0.67, 0.85]	3.5 [2.1, 5.9]	0.25 [0.10, 0.61]
**ERUS T4**	0.62 [0.25, 0.88]	0.98 [0.95, 0.99]	35.0 [11.3, 108.6]	0.39 [0.15, 1.02]
**MRI T4**	0.75 [0.37, 0.94]	0.95 [0.90, 0.97]	14.3 [6.0, 34.2]	0.26 [0.07, 0.91]

**Table 3:** Detailed areas under hierarchical summary receiver operating characteristic curve (hSROC) characterizing the accuracy of ERUS and MRI for the staging of T and N parameters.

	Area under ROC Curve [95% C.I.]	*p*-Value
**ERUS T**	0.91 [0.89–0.93]	0.409
**MRI T**	0.87 [0.84–0.89]	
**ERUS N**	0.92 [0.89–0.94]	0.389
**MRI N**	0.93 [0.90–0.95]	
**ERUS T1**	0.88 [0.85–0.91]	0.750
**MRI T1**	0.98 [0.96–0.99]	
**ERUS T2**	0.90 [0.88–0.93]	0.541
**MRI T2**	0.78 [0.75–0.82]	
**ERUS T3**	0.93 [0.91–0.95]	0.400
**MRI T3**	0.83 [0.79–0.86]	
**ERUS T4**	0.98 [0.97–0.99]	0.161
**MRI T4**	0.96 [0.93–0.97]	

## Data Availability

The data presented in this study are available on request from the corresponding Author.

## Supplementary Materials

The following supporting information can be downloaded at: https://www.mdpi.com/article/10.3390/diagnostics12010005/s1, electronic search strategy.
