# Supplementary material for: Endorectal Ultrasonography and Pelvic Magnetic Resonance Imaging Show Similar Diagnostic Accuracy in Local Staging of Rectal Cancer: An Update Systematic Review and Meta-Analysis
Source: Diagnostics (Basel). 2021 Dec 21;12(1):5. doi: 10.3390/diagnostics12010005 (PMC8775222; doi:10.3390/diagnostics12010005)
Supplement: Supplementary file 1 [file diagnostics-12-00005-s001.zip › diagnostics-1479048-supplementary.pdf]

(rectal cancer OR Rectal neoplasms OR rectal adenocarcinoma) AND (mri OR magnetic resonance imaging OR endorectal mri OR endorectal magnetic resonance imaging OR transrectal mri OR transrectal magnetic resonance imaging) AND (us OR trus OR erus OR eus OR ultrasonography OR endorectal ultrasonography OR transrectal ultrasonography OR endorectal-US OR transrectal-US) AND (staging) AND (accuracy).
